# Clinical analysis of prophylactic central neck dissection for papillary thyroid carcinoma

**DOI:** 10.1007/s12094-013-1038-9

**Published:** 2013-04-20

**Authors:** Q. Wang, B. Chu, J. Zhu, S. Zhang, Y. Liu, M. Zhuang, Y. Yang

**Affiliations:** Department of General Surgery, Xinhua Hospital, Shanghai Jiaotong University School of Medicine, Shanghai, 200092 China

**Keywords:** Thyroid carcinoma, Papillary, cN0, Central lymph node, Prophylactic, Central neck dissection

## Abstract

**Purpose:**

The need of prophylactic central neck dissection (PCND) in patients with papillary thyroid carcinoma (PTC) is still controversial. The major restriction of PCND is the potential complications. We undertook a retrospective study to discuss its necessity in PTC patients.

**Methods:**

A total of 188 patients with PTC who underwent total thyroidectomy and PCND were involved. In all of these, central lymph nodes were pathologic examined. Univariate and multivariate analyses were performed based on tumor location and size, etc.

**Results:**

Overall, node metastases were found in 44.1 % (83/188) of patients. Tumor size was the independent positive predictor for lymph node metastasis, while gender, age, tumor multifocality, tumor location, and capsular infiltration were not independent predictors of central lymph node metastases. Postoperative complications happened in 5.3 % (10/188) of patients, which 4.8 % (9/188) had temporary hypocalcemia and 0 % (0/188) had permanent hypocalcemia. Rates of temporary and permanent recurrent laryngeal nerve injury were 0.5 % (1/188) and 0 % (0/188), respectively.

**Conclusions:**

PCND is recommended in all patients with PTC.

## Introduction

Thyroid carcinoma is the most common endocrine malignancy, which incidence increased in the last several decades. Papillary thyroid carcinoma (PTC) is the most common histological subtype, accounting for more than 85 % of all cases. The prognosis of PTC is general good, with 10-year survival rate exceeds 95 % and 20-year survival rate exceeds 93 % [1–5]. Despite its excellent outcomes, studies have showed that recurrence is an important factor increasing in morbidity and mortality, and cervical lymph node metastasis is most important variable known to increase the risk of local recurrence [6, 7].

It has been shown that cervical lymph node metastasis was found in 20–90 % of patients who undertook cervical lymph node dissection [8, 9]. Of theses, central lymph node metastasis is most common [10, 11]. For now, routinely performing a neck dissection in patients with clinically positive neck lymph nodes (cN+) has been a consensus. However, there is a controversy concerning the need and indications for a neck dissection in PTC patients showing clinically negative neck lymph nodes (cN0), which means prophylactic central neck dissection (PCND) [12–16].

The present study was a retrospective analysis of patients with cN0 PTC from the General Surgery department of Xinhua Hospital and from the Renji Hospital affiliated to Shanghai Jiaotong University School of Medicine in the past 3 years. Univariate and multivariate analyses were performed to assess the factors predicting for central lymph nodes metastases. Results may provide clues on the necessity of PCND and contribute to the improvement of PTC treatment.

## Patients and methods

Subjects were selected according to the following criteria: (1) patients admitted for thyroid carcinoma in the General Surgery Department of Xinhua Hospital or in the Renji Hospital affiliated to Shanghai Jiao Tong University School of Medicine; (2) previously untreated patients; (3) surgical treatment was required for the primary tumor; (4) PTC diagnosed based on frozen paraffin sections; (5) PCND undertook in the same operating time after thyroidectomy; (6) lymph node metastases was not found clinically or radiologically prior to surgery (clinical N0/cN0); and (7) complete medical records were available for data extraction.

Following data were extracted from the medical records: gender, age at diagnosis, tumor size, tumor location, presence of local invasion, TNM classification, presence of central lymph node metastases, and postoperative complications.

TNM was assessed according to the 7th edition (2010) of the thyroid cancer TNM staging system from AJCC (American Joint Committee on Cancer)/UICC (International Federation of Cancer). The surgical procedure was followed according to NCCN practice guidelines. To the patients underwent total thyroidectomy we performed bilateral PCND, and to the patients underwent lobectomy the ipsilateral PCND was received.

Data collection was performed using Microsoft Excel (Redmond, WA, USA). Statistical analyses were performed using SAS 8.0 software (Cary, NC, USA). Student’s *t* test was used to compare normally distributed variables between groups. Non-normally distributed variables were analyzed using Wilcoxon’s rank test. Categorical variables were analyzed using the χ^2^ test or Fisher exact test. Multivariate analyses were conducted using unconditional logistic regression. Statistical significance was assumed when *P* < 0.05.

## Results

A total of 188 patients were involved in the present study. All cases were confirmed as PTC using frozen paraffin sections. Table 1 shows patient demographics. For each patient, an average of three lymph nodes were obtained (range, 1–16, median = 3). Pathological examination showed that central lymph node metastases were found in 83 patients (83/188, 44.1 %).

**Table 1:** Clinical and pathological characteristics of 188 patients with PTC

	No. of patients	%
Gender		
Male	35	18.6
Female	153	81.4
Age at diagnosis		
<45	83	44.1
≥45	105	55.9
Diseased lobe		
Left	71	37.8
Isthmus	2	1.1
Right	82	43.6
Left and right	33	17.6
Tumor size*		
≤1 cm	94	50
>1 and ≤2 cm	76	40.4
>2 and ≤4 cm	17	9.0
>4 cm	1	0.5
Multifocality		
No	144	76.6
Yes	44	23.4
Capsular invasion		
Yes	21	11.2
No	167	88.8
Central Lymph node metastasis		
Yes	83	44.1
No	105	55.9
Postoperative complications		
Yes	10	5.3
No	178	94.7
Tumor location**		
Up	20	14.1
Up-Middle	9	6.3
Middle	75	52.8
Middle-Down	19	13.4
Down	19	13.4
TNM group		
I	135	71.8
II	4	2.1
III	43	22.9
IV A	6	3.2
IV B	0	0.0
IV C	0	0.0

* Maximum measurement of tumor size

** Only in cases of solitary tumor located in left or right lobe

Postoperative complications happened in 10 patients (10/188, 5.3 %), in which 9 (9/188, 4.8 %) had temporary hypocalcemia and 0 (0/188, 0 %) had permanent hypocalcemia. Rates of temporary and permanent recurrent laryngeal nerve injury were 0.5 % (1/188) and 0 % (0/188), respectively.

Table 2 shows the results of univariate analyses based on central lymph node metastasis. Results revealed that large tumor size (*P* = 0.04), disease affecting both lobes (*P* = 0.04), and multifocality (*P* = 0.02) were significantly positively associated to central lymph node metastasis.

**Table 2:** Univariate analysis of central lymph node metastases

	Central Lymph node metastasis				*P*
	+ (%)		− (%)		
Gender					
Female	65	42.5	88	57.5	0.3364
Male	18	51.4	17	48.6	
Age at diagnosis					
<45	43	51.8	40	48.2	0.0601
≥45	40	38.1	65	61.9	
Diseased lobe 1					
Both	20	60.6	13	39.4	0.1096
Left	28	39.4	43	60.6	
Isthmus	0	0.0	2	100.0	
Right	35	42.7	47	57.3	
Diseased lobe 2					
Single	63	40.6	92	59.4	0.0360
Both	20	60.6	13	39.4	
Tumor size					
≤1 cm	33	35.1	61	64.9	0.0392
>1 and ≤2 cm	41	53.9	35	46.1	
>2 and ≤4 cm	8	47.1	9	52.9	
>4 cm	1	100.0	0	0.0	
Multifocality					
No	57	39.6	87	60.4	0.0226
Yes	26	59.1	18	40.9	
Capsular invasion					
No	75	44.6	93	55.4	0.6926
Yes	8	40.0	12	60.0	
Postoperative complications					
No	78	43.8	100	56.2	0.4733
Yes	5	50.0	5	50.0	
Tumor location					
Up	9	45.0	11	55.0	0.8645
Up-Middle	3	33.3	6	66.7	
Middle	30	40.0	45	60.0	
Middle- Down	9	47.4	10	52.6	
Down	6	31.6	13	68.4	
Total	83	44.1	105	55.9	

Meanwhile, gender, age at diagnosis, tumor size, multifocality, tumor location, and capsular invasion were put in the logistic regression model. Results show that tumor size was independent predictive factor for central lymph node metastasis (Table 3).

**Table 3:** Logistic regression analysis of central Lymph node metastases

	*Β*	SE	*P*	OR	95 % CI
Tumor size	1.2031	0.4159	0.0038	3.330	1.474–7.525
Constant	0.5720	1.4491	0.6930		

*SE* standard error, *OR* odds ratio, *CI* confidence interval

## Discussion

There is controversy in the application of PCND to the patients with PTC, which mainly focus on the balance of the potential benefits and the postoperative complications.

The benefits of PCND have become more and more clear. In our study, the positive nodes were found in 44.1 % of patients of PTC. Statistic analysis showed that the larger the tumor was, the more likely to find central lymph node metastasis, with the incidence of 35.1, 53.9, 47.1, and 100.0 % in tumor size of less than 1, 1–2, 2–4 cm, and lager than 4 cm, respectively. At the same time, tumor size was also an independent predictive factor for central lymph node metastases in cN0 PTC patients, with a positive correlation (correlation coefficient *β* = 1.2031, *P* = 0.004, OR = 3.330, 95 % CI = 1.474–7.525). These results consist with most reports. Ito et al. [14] also showed a positive correlation between tumor size and the incidence of central lymph node metastases, with the incidence of 38.3, 59.2, 73.7, and 79.0 % in tumor size of less than 1, 1–2, 2–4 cm, and lager than 4 cm, respectively. Roh et al. [12] using multivariate analyses reported that tumor size ≥1 cm was an independent factor for ipsilateral central lymph node metastases. Koo et al. [13] obtained the same results: either ipsilateral or contralateral lymph node metastases were more likely to occur in tumors >1 cm. However, a study by Kutler et al. [17] showed that tumor size had no significant impact on the presence of central lymph node metastases (*P* = 0.18). Generally, most researchers have had the consensus that the larger the tumor is, the more metastases may occurred.

This study also showed that both thyroid lobes had carcinoma would cause more nodal metastasis than one lobe, and multifocality of tumor would lead to higher incidence of metastasis than single tumor either.

As for the other factors such as patients’ age and gender, our results showed no significant difference in nodal metastasis. This consists with previously reported results [13, 17]. It means the application of PCND should not limited by patients age or gender.

Although not shown in this study, recent studies have shown that lymph node metastasis is related to increasing local recurrence and reducing survival rate. A large study have reported that mortality of PTC patients with nodal metastases was much greater than the patients without metastases. Thus to the patients with PTC, the benefits from PCND are obvious, the dissection can decrease local recurrence and increase the patient survival [6].

Another important reason to have PCND is that there is no other reliable method to verify central lymph node metastasis, whether preoperative or intraoperative ultrasound or other methods are far from sufficient sensitivity to verify the central lymph node metastasis [18]. Stulak et al. [19] showed in their study that in total of 511 patients with PTC, the clinical nodal negative patients were 476, in which preoperative ultrasound detected positive central lymph nodes in 10 patients (2.1 %) and positive cervical lymph nodes in 70 patients (14.4 %). But the truth was, the positive central lymph nodes were confirmed in 179 cases (32.5 %) and positive cervical lymph node was found in 86 cases (15.6 %). Kouvaraki et al. [9] showed that in 161 patients with preoperative ultrasonography, the positive central nodal incidence was 39.1 % (63/161), while the final pathological examination was proved to be 73.3 %, which implied false positive results in 2 (2/63) patients and 57 false negative results(57/98) of ultrasound examination. Therefore, before other reliable authentication methods arise, there is no better way to assess central lymph node metastasis other than PCND.

Furthermore, the results of PCND can help patients get clear staging after surgery, and subsequently take much more precise treatment. Explicit postoperative staging of patients with PTC can have following benefits of treatment: (1) to assess the prognosis; (2) to guide postoperative adjuvant therapy for individual programs, including ^131^I therapy and TSH suppressive therapy, which can reduce recurrence and mortality; (3) to determine the time and frequency of follow-up, which high-risk patients require intensive follow-up; (4) contribute to communication between physicians of patient information; and (5) staging system for clinical research can be used to assess the different groups of patients to different treatment [20, 21].

The main reason preventing us from taking PCND is the potential postoperative complications. As the increase in application of PCND, postoperative complications such as hypocalcemia, recurrent laryngeal nerve injury may increase [11, 22, 23] but as we have more experience, the incidence has been dropped obviously in the resent years [24–26]. Our study showed that the incidence of postoperative complications was 5.3 % (10/188); all of these were transient and no permanent complications, which included nine cases of hypocalcemia and one case of recurrent laryngeal nerve injury. But to the PTC patients who underwent recurrence, the reoperation for the treatment could be a total disaster. The disorders of structure would lead to much more severe complications and result in poor prognosis [27, 28].

In conclusion, the main reason stoping us taking PCND is the potential complications, but the data mentioned above have shown that after enough exercises, the incidence could be reduced to just like without PCND. Besides the high incidence of central lymph nodes metastasis in patients with PTC, the application of PCND will reduce tumor recurrence and patient’s mortality, and the pathological results of PCND will promote the follow-up treatment, we recommend PCND to all patients with PTC. But considering the good outcome of PTC and with the purpose to reduce complications as much as possible, we suggest that the prevention of complications should always be put to top priority in operation, which means during the surgery preference should be given to the removal of parts not leading to complications. After all, our purpose is to give patient better prognosis. In general, we recommend a compromised PCND to all patients with PTC, which means the PCND should compromise with the priority of low complication.
